# Concerns about the widespread use of rodent models for human risk assessments of endocrine disruptors

**DOI:** 10.1530/REP-13-0497

**Published:** 2014-04

**Authors:** René Habert, Vincent Muczynski, Tiphany Grisin, Delphine Moison, Sébastien Messiaen, René Frydman, Alexandra Benachi, Géraldine Delbes, Romain Lambrot, Abdelali Lehraiki, Thierry N'Tumba-Byn, Marie-Justine Guerquin, Christine Levacher, Virginie Rouiller-Fabre, Gabriel Livera

**Affiliations:** 1 Unit of Stem Cells and Radiation, Laboratory of Development of the Gonads, Sorbonne Paris CitéUniversité Paris DiderotBP 6, 92265 Fontenay-aux-RosesFrance; 2 CEA, DSV, iRCM, SCSR, LDG92265 Fontenay-aux-RosesFrance; 3 Unité 967INSERMF-92265 Fontenay aux RosesFrance; 4 Service de Gynécologie-Obstétrique et Médecine de la ReproductionHôpital A. Béclère, Université Paris SudF-92141 ClamartFrance; 5 INRS-Institut Armand FrappierLaval, QuebecCanada H7V 1B7

## Abstract

Fetal testis is a major target of endocrine disruptors (EDs). During the last 20 years, we have developed an organotypic culture system that maintains the function of the different fetal testis cell types and have used this approach as a toxicological test to evaluate the effects of various compounds on gametogenesis and steroidogenesis in rat, mouse and human testes. We named this test rat, mouse and human fetal testis assay. With this approach, we compared the effects of six potential EDs ((mono-(2-ethylhexyl) phthalate (MEHP), cadmium, depleted uranium, diethylstilboestrol (DES), bisphenol A (BPA) and metformin) and one signalling molecule (retinoic acid (RA)) on the function of rat, mouse and human fetal testis at a comparable developmental stage. We found that the response is similar in humans and rodents for only one third of our analyses. For instance, RA and MEHP have similar negative effects on gametogenesis in the three species. For another third of our analyses, the threshold efficient concentrations that disturb gametogenesis and/or steroidogenesis differ as a function of the species. For instance, BPA and metformin have similar negative effects on steroidogenesis in human and rodents, but at different threshold doses. For the last third of our analyses, the qualitative response is species specific. For instance, MEHP and DES affect steroidogenesis in rodents, but not in human fetal testis. These species differences raise concerns about the extrapolation of data obtained in rodents to human health risk assessment and highlight the need of rigorous comparisons of the effects in human and rodent models, when assessing ED risk.

## Introduction

In regulatory toxicology, the human health risk from exposure to a given endocrine disruptor (ED) is classically assessed using animal, especially rodent, models followed by an extrapolation of the data to humans. Generally, to define the regulatory acceptance for human health, the lowest no observed adverse effect level (NOAEL) measured in the rodent model is divided by a safety factor equal to ten to account for the difference between rodents and humans and by an additional tenfold uncertainty factor to account for inter-individual differences in susceptibility.

However, differently from fundamental processes such as cell differentiation, mitosis and carcinogenesis, many endocrine processes largely vary from one species to another. These species differences are even more pronounced for reproductive functions. Thus, the relevance of extrapolating animal data to human risk assessment should be questioned when assessing ED effects on reproductive functions.

The incidence of abnormalities of the male reproductive function has been increasing over the years (reviewed in Sharpe & Irvine (2004), Leridon & Slama (2008), and Main *et al*. (2010)). Human sperm count has been markedly decreasing and the rate of testicular cancer has clearly increased over the past four decades. Moreover, the prevalence rates of cryptorchidism and hypospadias are also probably increasing. More epidemiological, clinical and experimental data suggest that these male reproductive disorders could be due at least in part to the effects of EDs, which are becoming ever more concentrated and prevalent in our environment (Sharpe & Skakkebaek 1993, reviewed in Delbès *et al*. (2006) and Vandenberg *et al*. (2012)). Furthermore, it has been hypothesized that these alterations are symptoms of a single syndrome named ‘testicular dysgenesis syndrome’ (TDS), resulting from abnormalities in testis development during fetal life (Skakkebaek *et al*. 2001, Olesen *et al*. 2007). Thus, the fetal testis is a very relevant target to study the species differences in ED effects.

Fetal testis performs two major functions: gametogenesis and steroidogenesis (reviewed in Olaso & Habert (2000), Habert *et al*. (2001), and O'Shaughnessy & Fowler (2011)). Sertoli cells, which surround germ cells to form the seminiferous cords, are the first to differentiate at 11.5 days post-conception (dpc) in mice, 13.5 dpc in rats and 42–45 dpc in humans. Sertoli cells divide actively until puberty and then remain quiescent. In adult life, sperm production will depend on the number of Sertoli cells formed during this period (reviewed in Sharpe (2012)). In the early fetal testis, germ cells originated from migrating primordial germ cells, which are named gonocytes, proliferate. In rodents, they enter synchronously a quiescent period during which mitosis and apoptosis are arrested so that the number of gonocytes does not change (from 18.5 dpc to 3–4 days *post-partum* (dpp) in rats and from 15.5 dpc to 0–1 dpp in mice). In humans, from the beginning of the second trimester, gonocytes progressively differentiate into prespermatogonia (also called prospermatogonia or fetal spermatogonia) that no longer express C-KIT and OCT4 (Franke *et al*. 2004, Gaskell *et al*. 2004), similar to rodent germ cells during late neonatal life. These cells enter non-simultaneously a quiescent period for one group (Gaskell *et al*. 2004) whereas, for another group, some of these cells go on cell cycle (Pauls *et al*. 2006). Importantly, the differentiation of germ cells is not synchronous so that, even if each germ cell entered a quiescent period, the total number of germ cells per testis continuously increases during the whole fetal life in humans. As gonocytes give rise to the adult spermatogonial stem cells during neonatal life, correct development of the germ cell lineage during fetal/embryonic life is essential for the production of spermatozoa during adult life. Fetal Leydig cells differentiate soon after Sertoli cells and produce testosterone and insulin-like factor 3 (INSL3) that are required for the embryo phenotypic masculinization (reviewed in Scott *et al*. (2009) and Bay *et al*. (2011)).

In the 1990s, we developed an organotypic culture system, which we named fetal testis assay (FeTA; Habert *et al*. 1991, Lecerf *et al*. 1993, Olaso *et al*. 1998, Livera *et al*. 2000), that allows maintaining both gametogenesis and steroidogenesis of the rat fetal testis explants for a few days. We have adapted the system to mouse and human fetal testes over the past 10 years and this system allows a rigorous inter-species comparison of the fetal testis responsiveness and susceptibility to various chemicals. We review here data obtained for several EDs, the anti-diabetic drug metformin and retinoic acid (RA), a major paracrine/autocrine factor in fetal testis, covering a broad spectrum of genes and pathways potentially targeted by the chemicals and involved in toxicant-induced phenotypes.

## An experimental model to compare human, mouse and rat fetal testis responsiveness

### Choice of the system

**Figure 1:**
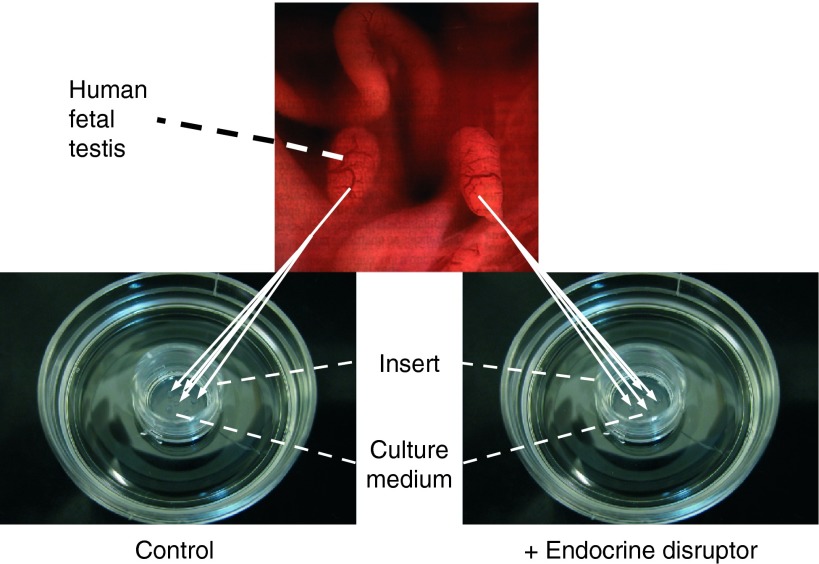
The fetal testis assay (FeTA). Human (6–12 GW), rat (14.5 dpc) or mouse (12.5 dpc) fetal testes are cultured on Millicell-CM Biopore membranes (pore size 0.4 μm, Millipore, Billerica, MA, USA) floating on 320 μl culture medium in tissue culture dishes at 37 °C in a humidified atmosphere containing 95% air/5% CO_2_. The culture medium is phenol red-free DMEM/Ham F12 (1:1) without biological factors and hormones. The culture medium is completely changed every 24 h. Whole mouse and rat testes are cultured on the membrane, while human testes are cut into small pieces (around 0.2 mm^3^) due to their larger size and three to four pieces are randomly placed on the membrane (two to eight wells per testis). The secretion of various molecules in the medium (testosterone, INSL3, AMH, transferrin, lactate, etc.) can be quantified every day. At the end of the culture, explants can be fixed and tissue sections can be used to assess, for instance, the total number of cells per testis, apoptotic activity (cleaved caspase 3, TUNEL assay) or mitotic activity (Ki67, BrdU incorporation), expression of Leydig cell (steroidogenic actors, INSL3, LH Receptors, etc.), Sertoli cell (AMH, SOX9, etc.) and germ cell markers (pluripotency genes, such as OCT3-4, NANOG and differentiation genes such as c-KIT, NANOS2 and DNMT3L).

**Figure 2:**
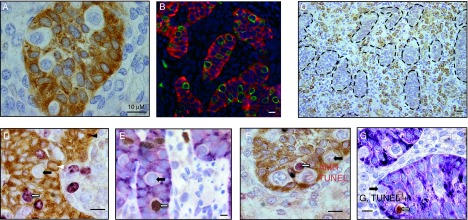
Immunohistological features of human fetal testis explants after culture. (A) AMH expression. In tissue sections from a ten GW human testis explant, Sertoli cells were immunostained with anti-AMH antibodies (brown cytoplasm). Gonocytes are identified as white cells inside the seminiferous cords. Bar: 10 μM. (B) Double immunofluorescence for AMH and M2A (9.5 GW human testis explants). M2A staining (green) is exclusively localized in gonocytes, characterized as large seminiferous cells that do not express AMH, a marker of Sertoli cells (red). Scale bar: 10 μM. (C) Cytochrome P450 scc expression. (9 GW human testis explant). Leydig cells are identified by cytochrome P450 scc expression. They are localized in the interstitial tissue between the seminiferous cords, which are here delineated by a dotted line. Scale bar: 20 μM. (D) Double immunostaining for AMH and Ki67 (7.5 GW human testis explant). Sertoli cells are identified by AMH expression (brown) and cycling cells are Ki67-positive (purple). Black arrow: Ki67-negative gonocyte. White arrow: Ki67-positive gonocyte. White arrowhead: Ki67-negative Sertoli cell. Black arrowhead: Ki67-positive Sertoli cell. Scale bar: 10 μM. (E) Double immunostaining for AMH and BrdU (10.7 GW human testis explant). BrdU was added 2 h before the end of the culture. Sertoli cells are identified by AMH expression (purple) and cycling cells in S phase are detected by immunostaining for BrdU (brown). Black arrow: BrdU-negative gonocyte. White arrow: BrdU-positive gonocyte. Scale bar: 10 μM. (F) Double immunostaining for AMH and TUNEL (11.5 GW human testis explant). Sertoli cells are identified by AMH expression (brown cytoplasm) and gonocytes as cells with white cytoplasm inside the seminiferous cords. Apoptotic cell are identified by TUNEL assay (brown nucleus). Black arrow: TUNEL-negative gonocyte. White arrow: TUNEL-positive gonocyte. Scale bar: 10 μM. (G) Double immunostaining for AMH and cleaved caspase 3 (ten GW human testis explant). Sertoli cells are identified by AMH expression (purple cytoplasm) and gonocytes as cells with white cytoplasm inside the seminiferous cords. Apoptotic cell are identified by immunostaining for cleaved caspase 3 (brown nucleus). Black arrow: caspase 3-negative gonocyte. White arrow: caspase 3-positive gonocyte. Scale bar: 10 μM.

One simple ethically acceptable way to evaluate directly the effect of a chemical compound in the human species is the use of *in vitro* approaches. However, the main limitation of toxicological studies using *in vitro* systems is the inability to reproduce the *in vivo* cell activities and fates. For instance, in cell culture systems, fetal Leydig cells dedifferentiate in the absence of specific gonadotropic stimulation (Habert & Brignaschi 1991, Rouiller-Fabre *et al*. 1998) and isolated gonocytes do not survive well (Boulogne *et al*. 2003). Organ culture systems in which the testis architecture and intercellular communications are preserved appeared to us to be a relevant method to maintain the development of fetal or neonatal testis. In the 1990s, we compared different *in vitro* systems and found that the best functional and histological results were obtained by placing rat fetal testis explants on a membrane that floats on the culture medium at the air–medium interface (Habert *et al*. 1991). This organotypic culture system allows reproducing *in vitro* the normal development of testis somatic and germ cells without addition of any exogenous signalling factor (Habert *et al*. 1991, Lecerf *et al*. 1993, Olaso *et al*. 1998, Livera *et al*. 2000). We then adapted this methodology also to the culture of mouse and human fetal testes (Lambrot *et al*. 2006*a*, Livera *et al*. 2006). We then validated this organotypic culture system under the name of rat, mouse and human FeTA (r/m/h FeTA) as a tool to study the age-, time- and dose-dependent direct effects of various compounds on fetal testis functions and development (Lambrot *et al*. 2006*b*). This organotypic culture system has also been used by others for rat (Cupp & Skinner 2001, Li & Kim 2003, Stroheker *et al*. 2006, Chauvigné *et al*. 2009) and human testis explants (Bendsen *et al*. 2001, Robinson *et al*. 2003, Hallmark *et al*. 2007). Recently, we also showed that the r/m/h FeTA assay is a valuable toxicological assay by demonstrating that the *in vivo* effects of mono-2-ethylhexyl phthalate (MEHP) during mouse fetal testis development could be reproduced in our culture system (Muczynski *et al*. 2012). The hFeTA assay and the histological features of human fetal testis explants at the end of the culture period are presented in Figs 1 and 2.

### When to do the explants?

Using this culture system, we observed clear age-related changes in the fetal testis responsiveness to various compounds. For instance, gonadotropin-releasing hormone (GNRH), a putative paracrine testicular factor, does not have any effect on testosterone secretion in 14.5 dpc rat fetal testes, whereas it has negative effects in 16.5–18.5 dpc testes and positive effects in testis explants from 20.5 dpc onwards (Habert *et al*. 1991). Oestradiol and diethylstilboestrol (DES) reduce testosterone production in 14.5 dpc rat fetal testis explants, but not in older specimens (Delbès *et al*. 2007). Similarly, the development of the germ cell lineage is affected differently as a function of age. MEHP apoptotic effect on gonocytes is much stronger in 11.5–13.5 dpc mouse fetal testis explants than in neonatal ones (Lehraiki *et al*. 2009). Age-related changes in the gonocyte response to various factors (RA, triiodothyronine, phorbol ester) have also been observed in cultures of dispersed testicular cells (Boulogne *et al*. 2003). Thus, to compare different species, it is necessary to choose a similar stage of development. We obtained human fetal testis samples from abortions carried out between the 6th and the 12th gestational week (GW), which corresponds to a period when testosterone secretion is increasing and gonocytes are proliferating (Rouiller-Fabre *et al*. 2009). To compare age-matched fetal testes from the three species, we decided to use 14.5 dpc rat (Habert & Picon 1984, Boulogne *et al*. 1999) and 12.5 dpc mouse testes (Vergouwen *et al*. 1991, Livera *et al*. 2006).

### Choice of culture conditions

Steroidogenesis can be measured using two parameters: i) the basal secretion of testosterone (i.e. in the absence of placental leutenizing hormone (LH) or human chorionic gonadotropin, hCG) that, in short-term cultures, reflects the *in vivo* testis activity (Habert & Picon 1982) and ii) the secretion of testosterone in the presence of LH/hCG to measure the explant maximum steroidogenic capacity. *In utero* decapitation of 16.5–18.5 dpc rat fetuses, a surgical operation that suppresses LH in fetal plasma and that is compatible with embryo survival, reduces basal but not LH-stimulated testosterone secretion *in vitro*, showing that endogenous LH is necessary for basal *in vivo* steroidogenic activity but not for the differentiation of Leydig cells (Migrenne *et al*. 2001). Moreover, in 18.5 dpc rat testis explants, addition of a GNRH agonist increases the basal secretion and decreases the LH-stimulated secretion of testosterone (Habert *et al*. 1991). Thus, when comparing the effect of a compound in different species, it is important to use the same parameters (basal or LH-stimulated testosterone secretion).

### Strengths and limits of the FeTA

The advantages of the organotypic culture system to study the chemical effects are numerous. This method allows the investigation of direct effects of chemicals alone or in mixture upon the fetal testis development and function. It allows a precise study of their kinetics of action and the duration and level of exposure is precisely controlled. These cultures are carried out in a defined medium (usually with no phenol red), thus avoiding cross-contamination with other biologically active molecules. The treated testicular explants are compared with explants issued from the same testis (when the fetal testes are large enough to be cut into different pieces, i.e. with human or older rodent testes) or with the contralateral testis from the same fetus (for 14.5–15.5 dpc rat and 12.5–13.5 dpc mouse fetuses) that are cultured with vehicle only and served as controls. This paired analysis limits individual variability and increases the sensitivity of the method. As pregnant rodent females can produce up to 15 embryos, it is possible to assay various doses or different compounds with a single pregnant female, thus reducing the number of animals killed in comparison with *in vivo* approach. FeTA is also largely less time- and labour-intensive and cheaper than *in vivo* approaches. FeTA is a convenient model to investigate the mechanism of action of ED. The use of fetal testes from transgenic mice easily allows the identification of the pathway involved in chemical effects. As an example, unlike the DES effect, the negative effect of bisphenol A (BPA) on testosterone produced by the WT mouse fetal testis was maintained when using an ERαKO fetal testis. This suggests a specific signalling pathway for BPA, which does not involve ERα and differs from the DES mode of action (Delbès *et al*. 2005, N'Tumba-Byn *et al*. 2012). Interestingly, such an experimental approach allows the use of transgenic mice in which knocking out the gene is lethal during gestation. Indeed, this method allows the maintenance of the development of the testis even beyond the death of the fetus (Petre-Lazar *et al*. 2007). Lastly, as shown in this paper, FeTA brings a valuable help in the translation of animal data to human beings.

As with each model, FeTA also has inherent limits. The main one is that long-term effects cannot be studied using this system as the development of the testis is maintained *in vitro* only for a few days (∼4–10 days depending on the species, the stage at sampling and the studied endpoint). Especially, this drawback is significant for the overall study of fetal gametogenesis in the human species that is a slow process. Xenograft of human fetal testes into immunodeficient rodent allows such long-term studies (Mitchell *et al*. 2010). However, for human fetal testicular steroidogenesis, there is no advantage in using the xenograft model in comparison with organotypic culture as the former is highly more time-, money- and animal-intensive and displays a higher variability than the latter. Furthermore, the metabolism of the chemicals in the rodent host can differ from that in humans. FeTA is a system that does not permit the study of delayed effect such as abnormal spermatogenesis. Such long-term effects that may result from alterations of the differentiation of the gonocytes that are the precursors of spermatogonia stem cells must be studied *in vivo*. Another drawback of the *in vitro* approach is that this method only allows study of the direct effect without considering the potential extra testicular feedback loops that could compensate the effects of exogenous chemicals. Moreover, it does not take into account the indirect effect that these molecules can exert, for instance via a change in the activities of the placenta or of the hypothalamo–hypophysis axis that are known to play a role in testis development.

## Species-dependent effects of various chemical compounds on fetal testis function

**Figure 3:**
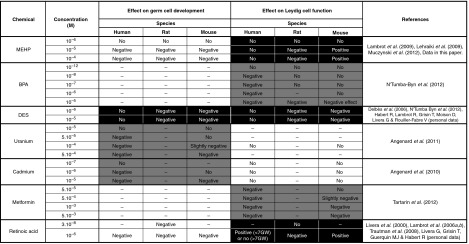
Comparison of the effects of seven chemicals on gametogenesis and steroidogenesis in human, rat and mouse fetal testis. Testes from 6 to 12 GW (human), 14.5 dpc (rat) or 12.5 dpc (mouse) fetuses were cultured for 3 or 4 days as described in the legend of Fig. 1. The culture medium, which did not contain any biological factor or hormone, was completely changed every 24 h. Germ cell development was assessed at the end of the culture period by quantifying the total number of gonocytes per testis, the apoptotic rate (percentage of TUNEL-positive or caspase 3-positive gonocytes) and the mitotic rate (percentage of gonocytes that incorporate BrdU added in the medium for the last 2 h of culture). Leydig cell function was assessed by quantifying the daily secretion of testosterone. No: not detectable effect; negative: the chemical reduces the number of gonocytes (and/or increases the apoptosis and/or decreases the mitotic rate) or decreases testosterone production; positive: the chemical increases the number of gonocytes (and/or decreases the apoptosis and/or increases the mitotic rate) or increases testosterone production; –: not performed. White area: the response is similar in human and rodent testes. Grey area: the response is qualitatively similar in human and rodent testes, but the NOAEL is different. Black area: the response qualitatively differs between human and rodent testes.

Using the r/m/h FeTA system, we compared the effects of seven compounds on gametogenesis and steroidogenesis in rat, mouse and human fetal testes explants. Results are summarized in Fig. 3.

### Phthalates

**Figure 4:**
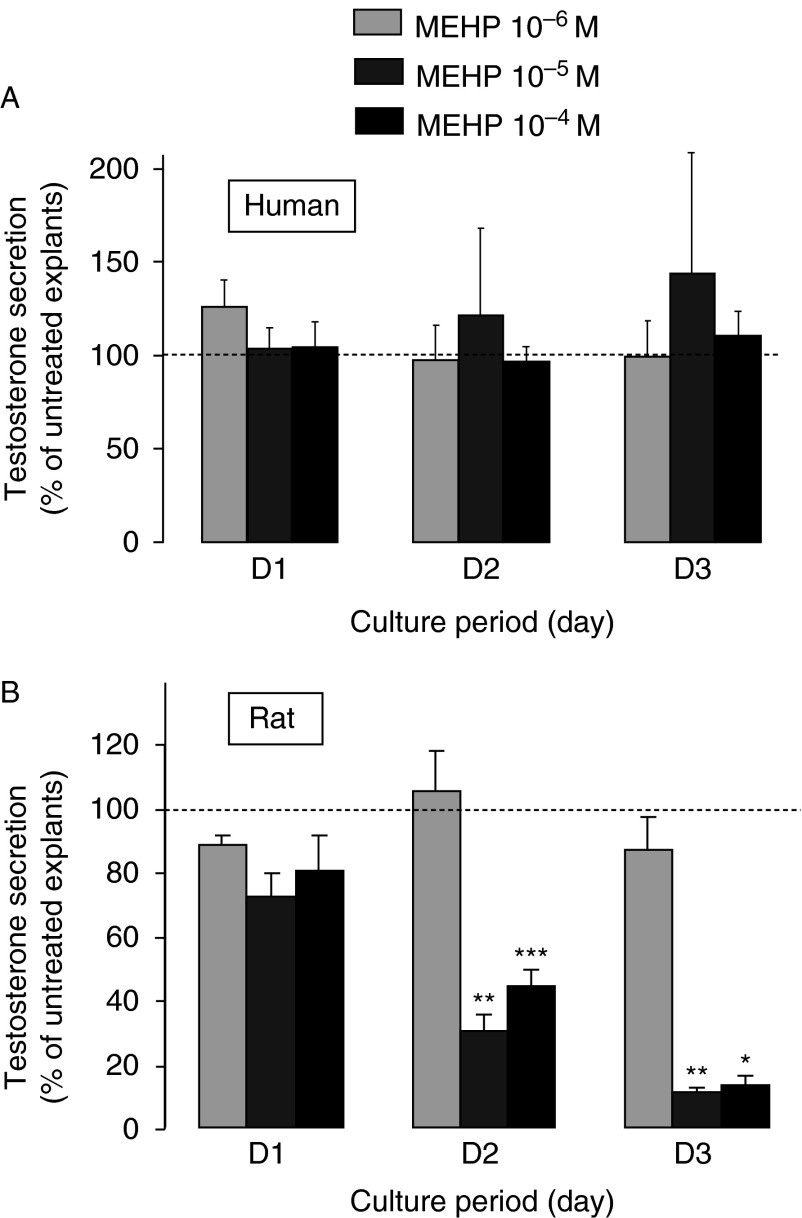
Effect of MEHP on testosterone secretion by cultured rat and human fetal testis explants. Testes from 7 to 11 GW human fetuses (A) to 14.5-day-old rat fetuses (B) were cultured using the FeTA system, a method in which the explants are deposited on floating membrane at the interface between air and medium that we had previously developed for the rats (Habert *et al*. 1991, Lecerf *et al*. 1993) and humans (Lambrot *et al*. 2006*a*,*b*). This method is briefly described in the legend of Fig. 1. Culture medium, which did not contain biological factors or hormones, was completely changed every 24 h. For each fetus, after 24 h of culture in control medium (D0), one testis was cultured in the absence (untreated) and the other one in the presence of MEHP at concentrations ranging from 10^−6^ to 10^−4^ M for 3 days (D1–D3). The daily testosterone secretion was measured by radioimmunoassay and the values at D1–D3 were normalized to the D0 secretion of the same testicular explant. Values (mean±s.e.m.) were then expressed as the percentage of the normalized secretion of the treated explant compared with that of the untreated explant. For human samples, *n*=3 for 10^−6^ M, *n*=3 for 10^−5^ M and *n*=15 for 10^−4^ M. For rat samples, *n*=5–7 for the three concentrations. *P<0.05, **P<0.01, ***P<0.001 compared with untreated testis using the Wilcoxon's non-parametric paired test. Values for human testis cultures are from Lambrot *et al*. (2009).

*In vivo* models have shown that phthalates impair gonocyte development in the rat (Barlow *et al*. 2003, Ferrara *et al*. 2006, reviewed in Habert *et al*. (2009)). Increased gonocyte apoptosis was also observed *in vitro* using the FeTA system in rat, mouse and human fetal testis explants (Chauvigné *et al*. 2009, Lambrot *et al*. 2009, Lehraiki *et al*. 2009). Conversely, MEHP induced the appearance of multinucleated gonocytes only in mouse and rat but not in human fetal testis explants. Moreover, MEHP did not affect testosterone production in human fetal testis explants (Lambrot *et al*. 2009). This was surprising because phthalates are considered anti-androgenic compounds based on their inhibitory action on *in vivo* production of testosterone in the rat (reviewed in Habert *et al*. (2009) and Scott *et al*. (2009)). One explanation could be that *in vitro* systems are unsuitable for detecting the anti-androgenic effects of phthalates. Indeed, some authors found that addition of MBP or MEHP to cultured rat fetal testes do not affect testosterone production (Stroheker *et al*. 2006, Hallmark *et al*. 2007). Conversely, MEHP increased or did not change the basal or LH-stimulated testosterone production by mouse fetal testis explants in the FeTA system (Lehraiki *et al*. 2009). However, another study reported a MEHP-induced reduction of testosterone secretion by fetal rat testes cultured in the FeTA system (Chauvigné *et al*. 2009). This effect could be observed when only half of the medium was changed daily, but not when the medium was completely changed every 24 h (i.e. the conditions we used for human testes in the study by Lambrot *et al*. 2009). This made us question our conclusion that phthalates are not anti-androgenic in the human species. Thus, we recently re-evaluated the effect of phthalates on testosterone production in rat fetal testes cultured rigorously following the same conditions as those we had used previously for human fetal testis (i.e. daily change of the whole medium). Original data are presented in Fig. 4B. We found that, unlike the results of Chauvigné *et al*. (2009), MEHP induced a dose- and time-dependent decrease in testosterone production in cultured rat fetal testis explants (Fig. 4). Thus, the rat model can be used as a positive control to validate the absence of phthalate effects on the steroidogenic response in human testis explants. Furthermore, we confirmed that MEHP increases germ cell apoptosis as the percentage of caspase 3-positive cells increased from 0.86±0.25 in controls to 8.14±0.13 in 10^−4^ M MEHP-treated testes at day 3 (D3) of culture. We cannot explain why Chauvigné *et al*. (2009) could not observe a steroidogenic effect of MEHP with a complete daily change of the medium. Nevertheless, this emphasizes that inter-species comparisons need to be carried out following a rigorous approach: i) selecting ages of gestation where the same events are occurring, ii) using an identical method across the different species that supports gametogenesis and steroidogenesis, iii) using explants with very similar size, iv) using explants that are not contaminated with mesonephros and v) reducing experimentation variability by using the same investigators and the same material.

The recent observations that di-*n*-butyl phthalate decreases steroidogenic activity in rat fetal testes but not in human fetal testes grafted into a host mouse or rat (Heger *et al*. 2012, Mitchell *et al*. 2012) definitively confirms that phthalates are not anti-androgenic compounds for human fetal testis.

### Bisphenol A

The FeTA system showed that the minimum BPA concentration required to impair testosterone production is at least 100-fold lower for human than for rat and mouse fetal testes (N'Tumba-Byn *et al*. 2012). Specifically, 0.01 μM BPA (i.e. a concentration relevant to human internal exposure) is sufficient to decrease testosterone production in human fetal testes, whereas concentrations as high as 1 or 10 μM are necessary in mouse and rat explants.

*In vivo* investigations of BPA effect upon fetal Leydig cell function led to contradictory conclusions. Exposure to high doses of BPA during pregnancy reduced plasma testosterone at birth in the rat (Tanaka *et al*. 2006). Administration of low doses (50 μg/kg per day) BPA to pregnant rats reduced the anogenital distance (AGD) in male pups, whereas lower BPA doses had no effect (Murray *et al*. 2007). To the contrary, three other independent studies did not show any effect of BPA on AGD after gestational administration of various doses from 1 to 50 000 μg/kg per day (Kobayashi *et al*. 2002, Tyl *et al*. 2002, Howdeshell *et al*. 2008). In humans, a recent retrospective epidemiological study highlighted that sons of workers who were professionally exposed to high levels of BPA during pregnancy had shorter AGD (Miao *et al*. 2011). However, no increase in BPA concentration in umbilical cord blood was observed in newborns with cryptorchidism (Fenichel *et al*. 2012).

### Diethylstilboestrol

The FeTA system showed that DES clearly impairs both steroidogenesis and gametogenesis in rat and mouse, but not human fetal testis explants (Delbès *et al*. 2007, N'Tumba-Byn *et al*. 2012 and Habert R, Lambrot R, Grisin T, Moison D, Livera G & Rouiller-Fabre V personal data). This is puzzling because it has been reported that *in utero* exposure to DES is associated with TDS (hypospermia, testicular cancer, hypospadias and cryptorchidism), although the data about the increased risk of testicular cancer following *in utero* DES exposure remain conflicting (reviewed in Delbès *et al*. (2006) and Mitchell *et al*. (2013)). The absence of DES effect on human fetal testicular steroidogenesis was confirmed recently using the xenograft model (Mitchell *et al*. 2013). This suggests that DES effects *in utero* on human testes concern endpoints that have not been yet studied, such as a direct effect on the genital tract or INSL3 secretion. Alternatively, it could impair the molecular differentiation of gonocytes in such a way to produce a delayed effect that can be detected only at later stages of development or in adult life. Further studies are needed to address this point.

### Depleted uranium

To our knowledge, no data on the *in vivo* effect of depleted uranium on the development of the fetal testis are available. The effect of non-radioactive uranium was assessed using the FeTA system only in human and mouse testis explants (Angenard *et al*. 2011). Uranium, even at high concentrations (100 μM), did not affect testosterone production in both species. Conversely, it decreased the number of gonocytes by inducing apoptosis. The NOAEL is between 10 and 50 μM for human testis and around 100 μM for mouse testis.

### Cadmium

The effects of cadmium were also compared only in the human and mouse species using the FeTA system (Angenard *et al*. 2010). Steroidogenesis was not affected by cadmium in both species, whereas the number of gonocytes was reduced by increased apoptosis. The NOAEL ranges were between 0.1 and 1 μM for human testis and between 1 and 10 μM for mouse testis. *In vivo*, gonadal development in mouse embryos exposed to cadmium in early organogenesis was studied by Tam & Liu (1985). Genital ridge size was reduced in exposed animals, with retarded germ cell migration into the ridges, resulting in depleted populations of germ cells, defective maturation of gametes and subfertility in male offspring.

### Metformin

Metformin is an anti-diabetic drug that is used to treat gestational diabetes and polycystic ovary syndrome. In the FeTA system, metformin reduces testosterone secretion in both human and mouse testis explants, but the NOAEL is <50 μM for human testis and around 500 μM for mouse testis (Tartarin *et al*. 2012). This result is important because the 50 μM level is in the range of concentrations measured in human plasma during therapeutic treatment. In agreement with the *in vitro* approach, daily administration of 300 mg/kg per day metformin to pregnant mice from 0.5 dpc induced a decrease in the fetal testicular testosterone content at 16.5 dpc (Tartarin *et al*. 2012).

### Retinoic acid

In the FeTA system, RA has a positive steroidogenic effect in human fetal testes (but only when younger than 7 GW) and in mouse fetal testes but a negative effect in rat fetal testes (Livera *et al*. 2000, Lambrot *et al*. 2006*a*, Livera G & Habert R personal data). Concerning gametogenesis, RA increases mitosis but also apoptosis (more strongly) in gonocytes; thus, the overall result was a decrease in the total number of gonocytes in cultured rat, mouse and human testis explants (Livera *et al*. 2000, Lambrot *et al*. 2006*a*, Trautmann *et al*. 2008, Guerquin MJ, Grisin T, Habert R & Livera G personal data). Specifically, RA induces meiosis in 12.5 dpc mouse explants, but not in 14.5 dpc rat explants and 6–12 GW human explants. This difference may not be only a consequence of a shift in developmental stage as the effect is retrieved with 11.5 dpc mouse testes but not in the 13.5 dpc rat testis. We rather favour the hypothesis that male commitment is governed by several pathways, the importance of which varies from one species to another. In agreement with our *in vitro* results, a mild vitamin A-deficient diet that results in a threefold decrease in plasma retinol concentration in pregnant rat increases the fetal testicular steroidogenesis at 18.5 dpc. This study showed that the endogenous retinol physiologically inhibits differentiation and/or function of fetal Leydig cells in the rat species (Livera *et al*. 2004). Similarly, the knockout of RA receptor α (RARα) results in an increase in the number of germ cells in mouse fetus (Livera G & Habert R personal data). Furthermore, RA prevents germ cell mitotic arrest both *in vitro* and *in vivo* (Trautmann *et al*. 2008).

## Conclusion

Over the past 10 years, we have compared the effects of six EDs and of RA in rat, mouse and human fetal testis explants using the r/m/h FeTA assay. The results show that these compounds can be classified into three groups based on the species-specific *in vitro* responses (Fig. 3). In the first group (with white background in Fig. 3), the *in vitro* response to a given compound is similar across the three species. In the second group (with grey background in Fig. 3), the response is qualitatively similar, but the NOAEL is different. For instance, the effect of cadmium and uranium on gonocyte development and the effect of BPA and metformin on steroidogenesis are qualitatively similar in three species, but the susceptibility is higher in humans than in rodents. These species differences probably result from differences in the expression of signalling pathways or endogenous hormonal production. Particularly, when considering the negative effect of BPA on steroidogenesis, its NOAEL is more than 100-fold lower in humans than in rodents. This raises concerns because the Sanitary Agencies routinely apply a security factor of 100 when extrapolating rodent data on BPA to human risk assessment. The third group (with black background in Fig. 3) includes compounds that show qualitative differences in the three species: phthalate effect on steroidogenesis, DES effect on gametogenesis and steroidogenesis and RA effect on steroidogenesis. This suggests that there are fundamental differences in the signalling pathways of phthalates, DES and RA in humans and rodents. For instance, the absence of DES effect in human fetal Leydig cells can be explained by the fact that ERS1 is not expressed in human fetal testis (Gaskell *et al*. 2003). More detailed studies must be performed to evaluate the endocrine differences and the various signalling pathways and molecular targets in human and rodent testes. Another surprising finding of this comparison is that some compounds, such as phthalates and RA, can have a stimulatory effect in mice and an inhibitory effect in rats. This may result from species differences in the rate-limiting steps of the steroid synthesis pathway or in the cellular localization of steroidogenic enzymes. Indeed, aromatase is mainly localized in Sertoli cells in rats and in Leydig cells in mice (Borday *et al*. 2013).

In conclusion, our findings obtained using the r/m/h FeTA assay show that the threshold of susceptibility to chemicals differs between human and rodent fetal testes for one third the tested compounds. Importantly, the very existence of a response, whatever the dose of the chemical, differs as a function of the species in another third of the cases. Depending on the chemical and on the biological function, its effect may be more intense or less adverse in human than in rodent testes. This raises concerns about the widespread use of rodent models to assess the adverse effects of EDs on human health. This study highlights the need to develop specific tools to study reprotoxicity specifically in humans and the importance of widening our knowledge on the molecular and endocrine differences between human and rodent fetal testis.

## Discussion from meeting

**Pia Juul Nielsen** (Copenhagen, Denmark): Are you indicating that you can use *in vitro* experimental studies to predict adverse effects, but you cannot predict the safe levels? You see responses in mice, rats and humans but at different dose levels.

**R Habert** (Fontenay aux roses, France): In our *in vitro* system named FeTA, the dose–response curve allows us to clearly define the no observed adverse level (NOAEL) and the lowest observed adverse effect level of one endocrine disruptor (ED) acting directly on the fetal testis. Obviously this is only one experimental approach and the FeTA results must be compared with other approaches to define the safe level in real life, which is a very difficult challenge. Importantly, if in the FeTA system, a given dose level of one compound shows no effect in humans, but effects are seen in rats and mice, or the opposite, it will be important to investigate the mechanisms of action of the compound in relation to species differences in its signalling pathway.

**Pia Juul Nielsen**: Your *in vitro* experiments with phthalates showed adverse effects in mice, rats and humans, therefore, it seems that these levels are not safe.

Richard Sharpe (Edinburgh, UK): There are differences depending on the endpoint assessed. Effects are variable for some endpoints (e.g. steroidogenesis), but there is more consistency when looking at the effects on germ cells. It is difficult to extrapolate from *in vitro* results to the *in vivo* situation, however.

**R Habert**: Using FeTA, we were very surprised to find that phthalates and DES did not reduce the production of testosterone by the fetal testis in human, which is opposite to the rat. Richard Sharpe *et al*. have also observed this recently using the xenograft approach. This is one additional validation of the *in vitro* approach.

**Niels E Skakkebæk** (Copenhagen, Denmark): Have you found greater variability in human cultures compared to animal cultures? There is much inconsistency in human populations, for example, we found a marked variation in quality of spermatogenesis in 5000 normal young men. Is there more variation in humans than animals? We would also expect variation in human fetal development.

**R Habert**: We observe a greater variability in human cultures than rodent ones. Humans being exposed to a variable environment whereas rodents have a uniform stable environment can explain this. Also, the method of preparing animal cultures is standardized but it is variable in humans. As an example, the delay between abortion and obtaining the fetal testes is variable. Lastly, there are many polymorphisms in human and not in pure rodent strains.

**Niels E Skakkebæk**: Although experimental procedures are as constant as possible, there must be inherent differences in the fetuses themselves, and rare cases could have Sertoli-cell-only testes.

**Richard Sharpe**: There is considerable variation in the level of testosterone production by xenografts of fetal testes from different human fetuses in Rod Mitchell's xenograft studies in nude mice. We do not have an obvious explanation for this between-fetus variation, but it is real. All of these were normal testes with germ cells.

**Ulla Hass** (Søborg, Denmark): There are species differences when comparing developmental toxicants in humans and animals. Substances should be evaluated as ED compounds (EDCs) if endocrine effects are seen in both rodents and humans, even if the specific effects are different.

**R Habert**: Species differences do not allow us to exclude phthalates as EDCs. Effects of phthalates largely change as the function of the species when considering the fetal Leydig cell function but they constantly reduce the number of gonocytes by increasing their apoptosis in rat, mouse and human. The rat model is relevant and important to human risk assessment when choosing a common effect in both species (such as gonocytes development when considering the phthalates). The recommended safe dose for humans is generally built by extrapolation from animal data using a safety factor equal to ten to account for species differences. However, the French National Heath Safety in Food, Environment and Work (ANSES) has recently used a factor for species differences equal to 30 to estimate the NOAEL for BPA.

**Luiz França** (Belo Horizonte, Brazil): In rats, there is a strong interaction between macrophages and Leydig cells. Does this have a role on the endocrine disrupting effects of phthalates?

**R Habert**: We have not examined the effects of EDCs on macrophages. Macrophages develop *in vivo* in the rat fetal testis, and 20 years ago we showed that the same development occurred in our *in vitro* culture system.

## Author contribution statement

V Muczynski, T Grisin, D Moison and S Messiaen performed the experiments for the original data presented here. V Muczynski, T Grisin, D Moison, S Messiaen, R Lambrot, A Lehraiki, T N'Tumba-Byn, M-J Guerquin, C Levacher and G Livera performed the experiments for personal data. V Rouiller-Fabre, A Benachi and R Frydman supervised the collection of human fetal testes. R Habert headed the group. G Delbes, V Rouiller-Fabre and G Livera contributed to critically reviewing the draft manuscript that was written by R Habert.
